# Supplementary material for: Chromothripsis during telomere crisis is independent of NHEJ, and consistent with a replicative origin
Source: Genome Res. 2019 May;29(5):737–49. doi: 10.1101/gr.240705.118 (PMC6499312; doi:10.1101/gr.240705.118)
Supplement: Supplemental Material [file supp_gr.240705.118_Supplemental_file_1.zip › contigs/annotated_contigs/DB57/contig.2.DB57_length_353_mean_cov_4.19830028329.docx]

**DB57_length_353_mean_cov_4.19830028329**

```
CCTAAATGAACTATGCTCTCTTTCAAGCCTCTGAGTCTTCCTACATGGTGTTTGCTCTCATCAGAATATGTATCCTCATCTTGTCAACA
 >chr7:147335637-147335814 - E=4e-89
AATGTTCTCTTCACACTGACCTTTTAGGCAAAATTATACCTTGCATTCTCTGCTATTGAAATGTTCTGTACATGTGGAATAAAGCAAG|

ATATC|TTATTTTAGATTTAGGGGGTATATGTGCAGGTTTGTTACCTGAGTAAATTGCATATTGTGGGGGGTTTGACATACAGATTATT
     >chr7:144664055-144664226 - E=1e-88
TCATCACCCAGGTAATGAGCATAATACCTGATAGGTATTTTCTGATTCTCACCCTCCTCCCACCCTTCACCCTCAAGTAGGCCTCAGT
```
